# Effects of Fish Oil with Heat Treatment on Obesity, Inflammation, and Gut Microbiota in Ovariectomized Mice

**DOI:** 10.3390/nu17030549

**Published:** 2025-01-31

**Authors:** Rong Fan, Judy Kim, Young-Cheul Kim, Soonkyu Chung

**Affiliations:** Department of Nutrition, University of Massachusetts, Amherst, MA 01003, USA; rongfan@umass.edu (R.F.); judykim@umass.edu (J.K.); yckim@nutrition.umass.edu (Y.-C.K.)

**Keywords:** hyperthermia therapy, synergy with heat and fish oil, microbiome, core body temperature, insulin sensitivity

## Abstract

**Background/Objectives:** Menopause induces substantial metabolic changes, including a reduction in metabolic rate and an elevated risk of developing metabolic diseases. Fish oil (FO) supplementation has been shown to ameliorate menopause-associated metabolic risks. Hyperthermia treatment (HT) has recently gained attention for its potential to improve metabolic and immune health. However, it remains to be determined whether HT can confer metabolic benefits comparable to those of FO supplementation or enhance the metabolic benefits of FO supplementation. This study aims to delineate the distinctive and collaborative effects of HT and FO supplementation in mitigating menopause-associated metabolic dysfunction. **Methods:** Female C57BL/6 ovariectomized (OVX) mice were randomly assigned to four groups (n = 12/group) to evaluate the individual and combined effects of FO supplementation (5% *w*/*w*) and HT treatment. For HT, whole-body heat exposure was conducted at 40–41 °C for 30 min, 5 days per week. After 12 weeks, animals were used to evaluate the changes in glucose and lipid metabolism, obesity outcome, and inflammatory markers. The gut microbiome analysis was conducted from cecal content by 16S rRNA sequencing. Acute inflammation was induced by lipopolysaccharide (LPS) injection to evaluate inflammatory responses. **Results:** HT alone distinctively reduced weight gain, lowered core body temperature, and attenuated insulin resistance comparable to FO supplement in OVX mice. The collaborative effect of FO and HT was not evident in metabolic parameters but more prominent in attenuating proinflammatory responses and microbiota modulation. **Conclusions:** Our findings suggest that the combined treatment of FO supplementation and HT may serve as an effective strategy to mitigate menopause-associated immune susceptibility and metabolic dysfunction. These benefits are likely mediated, at least in part, through the reduction in inflammation and modulation of the gut microbiota.

## 1. Introduction

Aging influences metabolism and overall health significantly. The risk and prevalence of obesity and metabolic dysfunction progressively become more common as people age, making it a critical public health concern. Menopause leads to hormonal shifts and physical alterations that have a substantial impact on metabolic health and raise the risk of metabolic disorders in individuals who are middle-aged or older [1,2]. This continuous, progressive decline is characterized by a significant escalation in metabolic dysfunctions between the ages of 45 and 50 [3,4].

The incidence of obesity and metabolic diseases increases significantly with reduced estrogen levels during the menopausal and post-menopausal stages [5,6]. Numerous studies have revealed that estrogen plays a vital role in regulating inflammation, insulin sensitivity, and metabolic rates [7,8,9]. Hormone replacement therapy has been used to alleviate menopause-related metabolic dysfunction. However, hormone replacement therapy is not always practical due to increased risks of certain cancers and cardiovascular complications [10,11]. Hence, novel therapeutic avenues are needed to provide safe and effective strategies to combat menopause-mediated metabolic dysfunction.

Interventions in diet and lifestyle are crucial for managing the metabolic challenges associated with menopause. Fish oil (FO) supplementation, which is abundant in n-3 polyunsaturated fatty acids, has been extensively studied for its anti-inflammatory properties, beneficial effects on lipid metabolism, and insulin sensitivity [12]. Thus, FO is a valuable dietary intervention for addressing the inflammatory and metabolic disturbances observed in post-menopausal women. Hyperthermia treatment (HT) has emerged as a promising intervention strategy to improve insulin sensitivity and mitochondrial function [13,14]. HT is a treatment that involves raising the body temperature, which can be applied either locally or to the entire body. The commonly used hyperthermia treatment practices include hot water immersion, infrared dry sauna, and wet/steam sauna [15,16,17,18]. The most commonly used temperature is around 40–42 °C, which is sufficient to induce physiological effects without causing injuries/damage, particularly to nervous tissue [19,20]. Unlike cold temperature-induced metabolic benefits achieved through activation of the sympathetic nervous system, HT offers a more manageable alternative with promising metabolic modulation benefits, particularly for elderly individuals [15]. This study aimed to determine whether HT is a lifestyle modification comparable to dietary FO intake and whether the combined treatment of HT with FO could produce a synergistic effect against menopause-mediated metabolic diseases such as diabetes.

The gut microbiota closely impacts energy homeostasis, host metabolism, and immunity. Diet and lifestyle are two main factors in shaping the gut microbiota composition and function [21,22,23]. Western diet has been shown to cause gut microbial dysbiosis, contributing to the etiology of obesity and associated metabolic syndromes [24]. Intriguingly, recent research has suggested that temperature is a potential modulator of gut microbiome composition and function. Housing rodents at a thermoneutral temperature (30 °C) or exposing them to a cold temperature (4 °C) induces distinct changes in gut microbial profiles compared to those housed at ambient temperatures (22–26 °C) [25,26]. Consistently, moderate physical activity has been linked to improved gut health and reduced susceptibility to intestinal inflammatory diseases [27]. Changes in core body temperature during and after regular physical activities are known to mediate metabolic adaptations, including modulations of gut microbiome composition [28]. Exploring alternative methods to regulate core body temperature independently of physical activity may offer a novel approach to achieving the metabolic benefits typically associated with exercise. This is particularly important for individuals who face challenges in engaging in regular physical activity.

This study aims to delineate the distinctive and collaborative effects of HT and FO supplementation in mitigating menopause-associated metabolic dysfunction using an ovariectomized (OVX) animal model. We focused on investigating the benefits of the combined treatment on insulin sensitivity, lipid metabolism, gut microbiota changes, and inflammation. Our results showed that FO and HT improve insulin sensitivity, alleviate acute inflammation, and restore metabolic health associated with menopause by reducing inflammation and modulating the gut microbiota.

## 2. Materials and Methods

### 2.1. Animals

All animal care and protocols were approved by the Institutional Animal Care and Use Committee (IACUC) of the University of Massachusetts Amherst (protocol 4081). A total of 48 five-week-old female C57BL/6 mice underwent bilateral ovariectomy (OVX) and were obtained from The Jackson Laboratory (Bar Harbor, ME, USA). The mice were acclimated for one week before the experiment. Animals were housed in a 12:12-h light: dark cycle in a specific pathogen-free barrier facility at 21–22 °C with 45% humidity control.

They were then divided into four groups (n = 12 per group, power of 80% with a *p*-value of 0.05, and a standard deviation of 10–15% differences in serum insulin levels) and randomly assigned to one of the following four experimental groups based on diet and HT (no confounders were controlled), CON: control diet, HT: CON diet with 30 min daily hyperthermia treatment (HT), FO: isocaloric diet containing fish oil, FOHT: FO diet with 30 min daily HT. Three mice were housed together, and all mice were fed an isocaloric diet ad libitum. The CON diet comprised 45% total fat, while the FO diet also contained 45% fat, with 10% replaced by fish oil from Sigma (Saint Louis, MO, USA, F8020, Menhaden fish oil, DHA: EPA = 1:1). The detailed diet composition is provided in Supplementary Table S1.

HT was conducted in an environment chamber (Power Scientific, Inc., Doylestown, PA, USA; humidity 50–70 ± 5% with an airflow rate of 10–15 air changes per hour) for whole-body heat exposure at 40–41 °C for 30 min/day, five days/week, for 12 weeks. We adopted a 30-min HT treatment based on evidence from previous studies [29,30]. Body weight and food intake were monitored weekly. A set of mice (n = 6 in each group) was used for the acute inflammation study via intraperitoneal injection of 50 μg lipopolysaccharides (LPS, Sigma-Aldrich, St. Louis, MO, USA). At the end of the study, all mice were deeply anesthetized using isoflurane. Whole blood was collected via retro-orbital bleeding in an EDTA-treated tube and subsequently centrifuged at 2000× *g* for 15 min at 4 °C to obtain plasma. After exsanguination, death was confirmed by cervical dislocation. Additionally, liver tissue was harvested and immediately snap-frozen in liquid nitrogen. Subsequently, all samples were stored at −80 °C until further analysis. Plasma levels of triglycerides (Abcam ab178780, Cambridge, MA, USA) and cholesterol (Abcam, ab65390 Cambridge, MA, USA) were determined according to the manufacturer’s instructions.

### 2.2. Metabolic Assessments

After overnight fasting (12 h), fasting glucose levels were measured from tail-bleeding using a glucometer, and fasting serum (centrifuged at 2000× *g* for 15 min at 4 °C) was also used to determine insulin levels using a mouse ultra-sensitive insulin ELISA (Crystal Chem 90080, Elk Grove Village, IL, USA). Homeostatic Model Assessment of Insulin Resistance (HOMA-IR) was calculated using fasting insulin and glucose. For the glucose tolerance test (GTT), glucose (1 g/kg body weight) was injected into the mice intraperitoneally after 6 h of fasting, and blood glucose was measured at 0, 15, 30, 60, and 120 min. At 30 min post-injection, blood was collected for insulin measurement. All animals had free access to water throughout the GTT. Core body temperature was measured by a rodent rectal probe (Kent Scientific Corp., Torrington, CT, USA).

### 2.3. RT-qPCR

Approximately 50 mg of the liver or ~100 mg of adipose tissues were homogenized in 1 mL of TRIzol and subsequently used for mRNA isolation. A total of 1 μg of mRNA was reverse transcribed to complementary (cDNA) using iScript™ Reverse Transcription Supermix (BioRad, Hercules, CA, USA). SYBR Green (BioRad, Hercules, CA, USA) supermix was used for qPCR. In detail, qPCR steps include an initial step of 3 min at 95 °C to activate Taq DNA polymerase, followed by 40 cycles of a 15-s denaturation at 95 °C and then 45 s of annealing at 60 °C and extension at 65 °C. The 2^−ΔΔCT^ method was used to determine the relative expression with normalization by housekeeping genes. Primer sequences are available in Supplementary Table S2.

### 2.4. 16S rRNA Sequencing and Microbiome Analysis

Cecal content was collected at the time of sacrifice, and genomic DNA was isolated using a Quick-DNATM Fecal/soil Microbe miniprep kit (Zymo Research, Irvine, CA, USA). 16S rRNA sequencing (n = 6 per group) was performed in the Genomic Resources Laboratory (GRL) at UMass Amherst. The microbial small subunit ribosomal RNA gene (16S v4 rRNA) was amplified using the primer set used by Earth Microbiome Project with slight modifications (CS1_515F, CS2_806R). The raw data were processed and analyzed on Unity Cluster at UMass Amherst using QIIME2 (version 2021.8) [31] to perform sequence denoising, dereplication, and chimera removal with the DADA2 plugin [32]. Detailed standards are as follows: quality filter Phred score > 30, truncate length 150 base pairs, and rarefaction to 28,910 sequences per sample (corresponding to the lowest sample read count).

Taxonomic classification and phylogenetic analysis were conducted by QIIME2’s pre-trained Naive Bayes classifier, using the SILVA reference database (version 138) with a 99% similarity threshold and q2-feature-classifier plugin. Subsequent analysis was carried out in R for alpha- and beta-diversity, analysis of relative taxonomic composition, and differential abundance analysis using dplyr, phyloseq, vegan, DESeq2, ggplot2, and pheatmap packages. Phyla with a median relative abundance > 0.1% and genera with a group median relative abundance > 1% were retained for relative abundance analysis.

The difference in alpha-diversity between different groups was determined by the Kruskal–Wallis test. Beta-diversity was measured by Bray–Curtis distance and presented as a principal coordinates analysis (PCoA) plot. Statistical difference was tested by Permutational multivariate analysis of variance (PERMANOVA) (999 permutations) for beta-diversity. Spearman’s rank correlation was used to show the correlation between bacterial genera and host metabolic factors. The coefficient ranges (−1,1); the negative coefficient indicated a negative correlation, and the positive coefficient indicated a positive correlation. Significance was set at *p* < 0.05 with post-hoc pairwise comparisons corrected for multiple testing using the Benjamini–Hochberg method.

### 2.5. Statistical Analysis

Statistical tests were performed by Graph Pad Prism (Version 8.0.2) and R studio (4.3.0). All data are presented as mean ± SEM. Independent samples from the control group and treatment group were analyzed and compared using two-tailed Student’s *t*-test, with * *p* < 0.05, ** *p* < 0.01, *** *p* < 0.001, or one-way ANOVA with Tukey’s multiple comparisons.

## 3. Results

### 3.1. HT Attenuated Body Weight Gain and Induced Metabolic Adaptation Without Causing Cell Damage Independent of FO Supplementation

**Figure 1:**
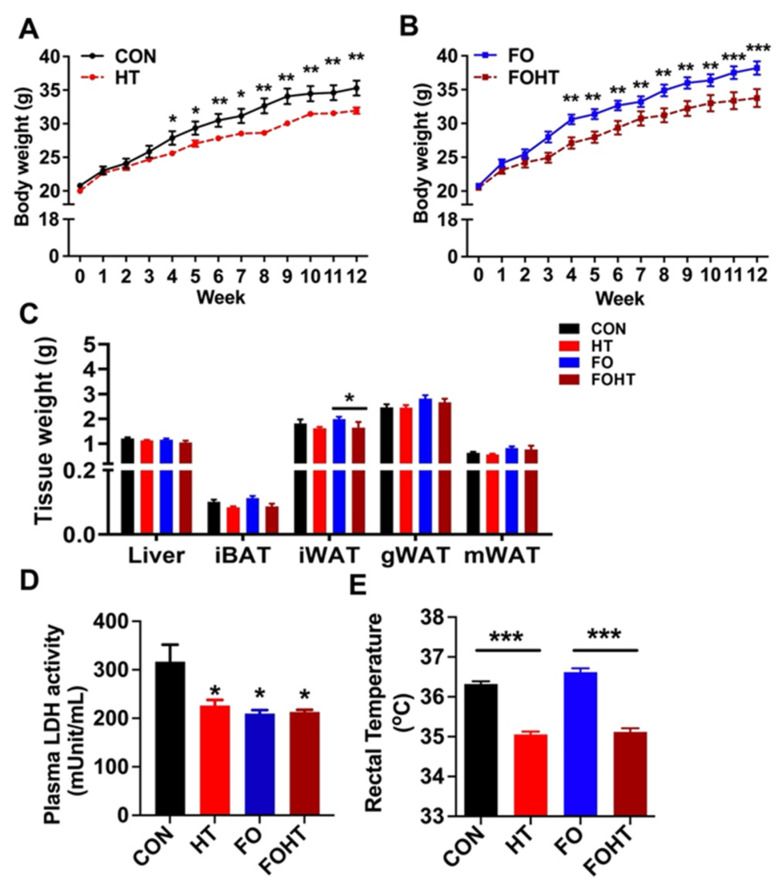
**FO and HT intervention significantly reduced body weight gain and induced metabolic adaptation.** Six-week-old OVX mice were fed either a CON or FO diet with or without a daily 30-min HT intervention for 12 weeks (n = 12/group). (**A**) Impact of HT on body weight gain in CON-fed mice. (**B**) Impact of HT on body weight gain in FO-fed mice. (**C**) Changes in tissue weights of the liver, interscapular brown fat (iBAT), inguinal (iWAT), gonadal (gWAT), and mesenteric white adipose tissue (mWAT). (**D**) Plasma levels of lactate dehydrogenase (LDH) activity. (**E**) The resting core body temperature was measured 24 h after the last HT session. All data are presented as mean ± SEM. (**A**–**E**) n = 12 per group. * *p* < 0.05; ** *p* < 0.01; *** *p* < 0.001 by Student *t*-test. **Abbreviations:** CON: Control diet (45% calories from fat), HT: CON diet and received with daily HT intervention for 30 min. FO: isocaloric diet containing 10% fish oil, FOHT: FO diet and received daily HT intervention for 30 min.

First, we assessed the metabolic impact of HT in OVX mice fed either a CON or FO diet. The daily application of HT intervention for 12 weeks (30 min per day) significantly reduced body weight gain in both the CON group (Figure 1A) and the FO group (Figure 1B). There were no significant differences in the tissue weights of the liver, gonadal fat, mesenteric fat, or brown fat among the groups; however, inguinal fat mass (iWAT) was significantly reduced in the FOHT group compared to the FO group alone (Figure 1C). To assess whether HT induces tissue damage, we measured plasma levels of lactate dehydrogenase (LDH) as a surrogate marker for cell or tissue damage. Compared to the CON group, both HT and FO significantly lowered plasma LDH levels, suggesting that neither HT nor FO caused tissue damage (Figure 1D). Conversely, this result revealed that both HT and FO interventions reduced cellular damage, although the synergistic effect of these two interventions was not evident.

Importantly, HT intervention significantly reduced the core body temperature independent of dietary intervention, implicating that HT alone posed a significant impact on metabolic adaptation (Figure 1E). These data suggested that FO and HT protected against weight gain and induced metabolic adaptation without causing cell damage in OVX mice.

### 3.2. HT Significantly Improved Insulin Sensitivity in OVX Mice, Comparable to FO Supplementation

**Figure 2:**
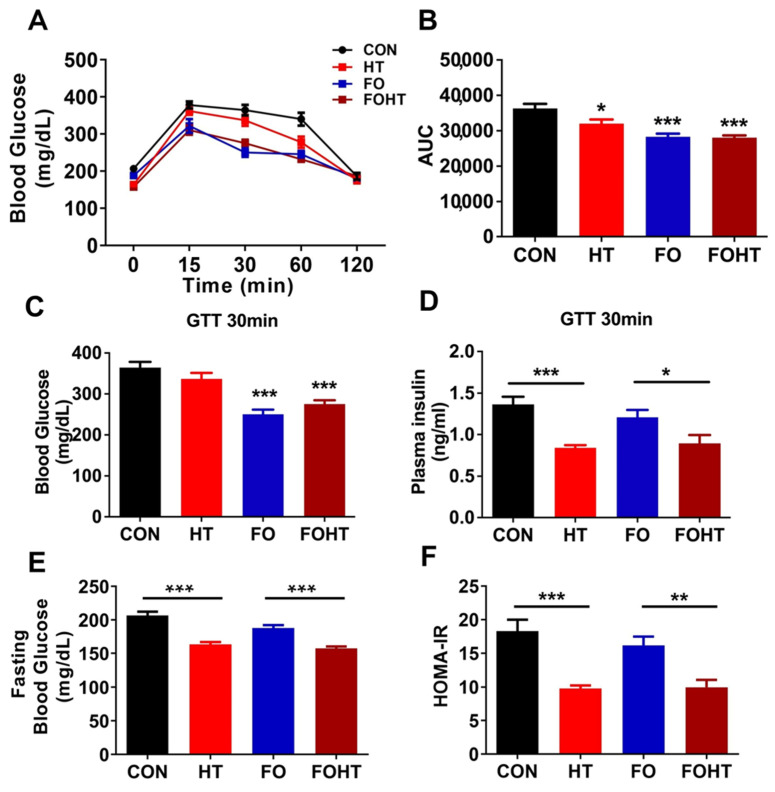
**HT intervention significantly improved insulin sensitivity both in CON and FO-fed OVX mice.** After 12 weeks of dietary intervention in combination with HT, OVX mice were subjected to a GTT test (n = 12 per group). (**A**) GTT, (**B**) Quantification of GTT by area under the curve (AUC). (**C**) Plasma glucose levels at 30 min during GTT. (**D**) Plasma insulin levels at 30 min during GTT. (**E**) Fasting blood glucose (mg/dL) (**F**) Homeostatic model assessment for insulin resistance (HOMA-IR). All data are presented as mean ± SEM. * *p* < 0.05; ** *p* < 0.01; *** *p* < 0.001 by Student’s *t*-test. **Abbreviations**: CON: Control diet (45% calories from fat), HT: CON diet and received with daily HT intervention for 30 min. FO: isocaloric diet containing 10% fish oil, FOHT: FO diet and received daily HT intervention for 30 min.

Next, we determined the impact of a potential synergy of HT and FO on insulin sensitivity using the OVX mice. Glucose tolerance test (GTT) results showed that HT induced a substantial increase in glucose disposal comparable to FO supplementation, while the synergy between FO and HT was not evident (Figure 2A,B).

At 30 min of GTT, there was no difference in the blood glucose levels between CON and HT, while plasma insulin levels were significantly reduced, which implies that HT promotes insulin sensitivity (Figure 2C,D). Consistently, FOHT further decreased the plasma insulin levels despite no changes in blood glucose levels (Figure 2C,D). The effect of HT in both CON and FO diets was potent in significantly reducing fast plasma glucose (Figure 2E, Table 1), and HOMA-IR, an index for insulin resistance, was significantly reduced (Figure 2F). Although the synergy between HT and FO in promoting insulin sensitivity was not evident, our work demonstrated that HT alone enhances insulin sensitivity in both CON and FO diets, even in young OVX mice.

### 3.3. FO and HT Synergistically Reduced Inflammation in the Liver of the OVX Mice

**Figure 3:**
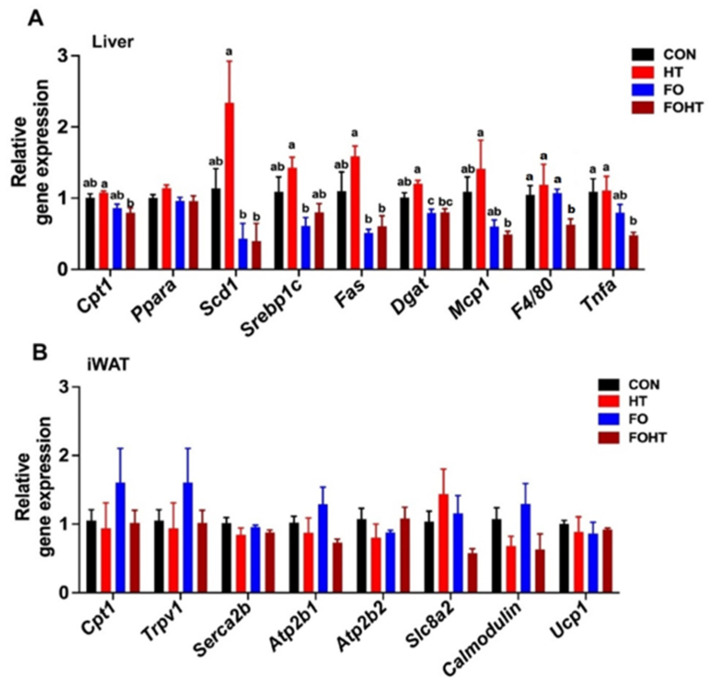
**The combined intervention of FO and HT exhibited a synergistic effect in attenuating inflammation in the liver but not in the iWAT of OVX mice**. After 12 weeks of dietary intervention in combination with HT, liver and iWAT tissues were collected for qPCR analysis. (**A**) Hepatic gene expression related to energy expenditure (Cpt1, Ppara), lipid synthesis (Scd1, Srebp1c, Fas, Dgat), and inflammation (Mcp1, F4/80, Tnfa). (**B**) Inguinal fat gene expression of fatty acid oxidation (Cpt1), uncoupling protein 1 (Ucp1), and Ucp1-independent thermogenic genes associated with futile calcium cycling (Trpv1, Serca2b, Atp2b1, Atp2b2, Slc8a2, Calmodulin). Data are presented as mean ± SEM (n = 5–6/group). Groups that do not share the same letter (a–c) are significantly different (*p*-value < 0.05) according to one-way ANOVA followed by Tukey’s multiple comparison post-hoc analysis. Abbreviations: CON: Control diet (45% calories from fat), HT: CON diet and received with daily HT intervention for 30 min. FO: isocaloric diet containing 10% fish oil, FOHT: FO diet and received daily HT intervention for 30 min.

Given the improved insulin sensitivity (Figure 2), we measured the metabolic impact of FO and HT on the liver and adipose tissue (only six animals were used since six animals were used for LPS stimulation). In agreement with the numerous studies showing the lipid-lowering effects of FO, FO significantly reduced lipid synthesis gene expressions, including the gene expression levels of stearyl CoA desaturase (*Scd1*), sterol regulatory element binding protein 1c (*Srebp1c*), fatty acid synthase (*Fas*) and diacylglycerol acyltransferase (*Dgat*) in the liver. In contrast to our expectation, there was no synergy between FO and HT in lowering the liver lipid in OVX mice (Figure 3A). Similarly, HT showed no significant impact on liver lipid levels in OVX mice. Intriguingly, however, combined treatment of FO and HT significantly reduced inflammatory cytokine expression levels of tumor necrosis factor-alpha (*Tnfa*) and macrophage marker *F4/80*. Also, there was a trend towards a decrease in proinflammatory marker of monocyte chemoattractant protein 1 (*Mcp1*) (Figure 3A). Next, we examined the separate and combined impact of HT and FO on energy expenditure-related genes in the inguinal fat of OVX mice. Neither FO nor HT altered energy expenditure-related gene expressions, including (1) fatty acid oxidation such as carnitine palmitoyltransferase 1(*Ctp1*), (2) uncoupling protein 1 (*Ucp1*), and (3) *Ucp1*-independent exergy expenditure-related genes via futile calcium cycling (Figure 3B). These results showed that HT or FO intervention had a minimal impact on the iWAT compared to the liver in OVX mice. More importantly, our work suggests that FO and HT could synergistically modulate hepatic transcription to attenuate inflammation in OVX mice.

### 3.4. FO and HT Altered Microbiota Diversity and Composition in OVX Mice

**Figure 4:**
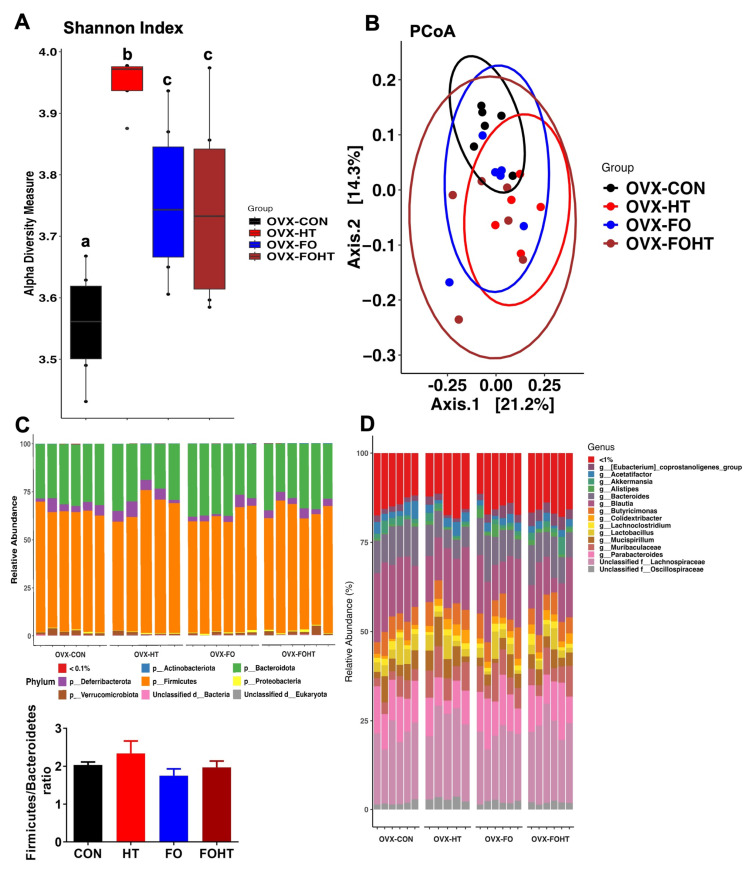
**HT and FO altered microbiota diversity and composition in OVX mice**. (**A**) Alpha-diversity (Shannon index). Statistical significance was determined at *p* < 0.05 and represented by different letters (a–c) by the Kruskal–Wallis test. (**B**) Beta-diversity by PCoA based on the Bray–Curtis index distance and permutational multivariate analysis of variance (PERMANOVA), (**C**) Stacked bar plots for phyla relative abundance, F/B (Firmicutes to Bacteroidetes) ratio. (**D**) Stacked bar plots for microbiota relative abundance at the genus level.

Next, we analyzed the gut microbiota to identify its potential contribution to metabolic activation by combining FO with HT in OVX mice. The 16S rRNA microbiome sequencing revealed that HT, FO, and the combination of FO and HT significantly increased alpha-diversity and Shannon index compared with CON (Figure 4A). To explore the between-sample diversity, we also assessed beta-diversity. The principal component analysis (PCoA) based on the Bray–Curtis distance did not show a clear separation among the four groups (Figure 4B), suggesting less between-sample diversity in the HT and FOHT groups compared to the CON and FO diets.

Next, we assessed the gut microbiota composition changes. The relative abundance plot (Figure 4C) revealed the phyla compositional difference between the four groups. Both HT and the combination of FO with HT did not significantly alter the *Firmicutes* to *Bacteroidetes* ratio (Figure 4C). The average relative abundance of *Firmicutes* is 62.6%, 65.5%, 60.6%, and 62.44% in the CON, HT, FO, and FOHT groups, respectively, while the average relative abundance of *Bacteroidetes* is 30.4%, 27.2%, 34.0%, and 30.8% in the CON, HT, FO, and FOHT groups, respectively. Interestingly, the average abundance for *Deferribacteres* is 4.2%, 5.2%, 3.4%, and 3.8%, respectively. The relative abundance at the genus level revealed the bacterial composition changes. (Figure 4D). In detail, HT increased *Muribaculaceae* from 3.7% in CON to 6.9% in HT (*p* < 0.05). The abundance of *Muribaculaceae* also increases in FO by 6.5% and 5.4% in FOHT compared to CON (*p* < 0.05). The average relative abundance of *Blautia* was measured at 16.5%, 14.9%, 14.4%, and 11.5% in the OVX-CON, OVX-FO, OVX-HT, and OVX-FOHT groups, respectively (n.s.). Similarly, an Unclassified *Lachnospiraceae* family showed relative abundances of 19.7%, 19.1%, 22.9%, and 22.2% across the groups (n.s.). The abundance of *Eubacterium coprostanoligenes* group increased by FO from 1.6% in OVX-CON to 2.5% in OVX-FO (*p* < 0.05) and 1.4% in OVX-HT and 3.3% in OVX-FOHT (*p* < 0.05). *A2* showed lower relative abundances, with 0.87%, 0.51%, 1.71%, and 0.89% across the groups (n.s).

Notable shifts in bacterial composition were observed in the OVX-HT and OVX-FOHT groups, particularly affecting *Blautia*, the Unclassified *Lachnospiraceae* family, and *Muribaculaceae*. Lastly, the beta-diversity of FO, HT, or the combination of FO and HT, did not have a similar effect in OVX mice compared to the non-OVX old mice, suggesting a potent impact of the absence of estrogen on microbiota (Figure S1A). For example, the relative abundance of an Unclassified *Lachnospiraceae* family was elevated in OVX mice, while *Bacteroides* showed a noticeable decrease compared to non-OVX mice. Similarly, *Muribaculaceae* increased in the OVX condition. Additionally, *Akkermansia* and *Parabacteroides* also displayed altered abundances, with the latter notably reduced in OVX mice. Variations were also observed in *Butyricimonas* and *Mucis spirillum* (Figure S1B).

### 3.5. FO and HT Significantly Protected Against LPS-Induced Acute Inflammation

**Figure 5:**
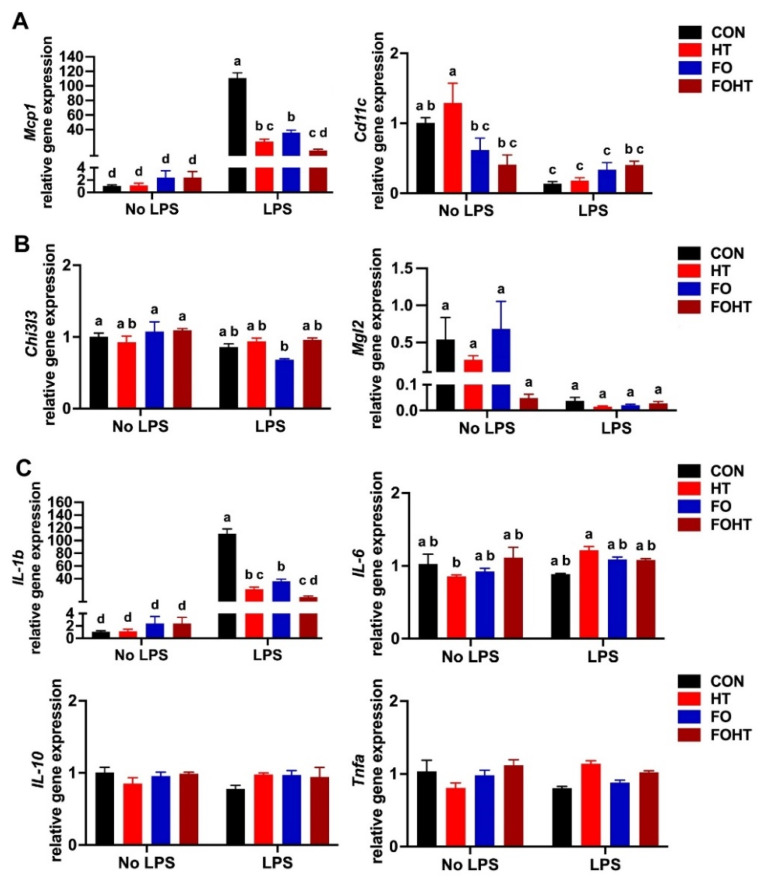
**FO and HT protected against LPS-induced acute inflammation**. (**A**) Peritoneal macrophage gene expression of inflammatory macrophages marker: Mcp1, and Cd11c. (**B**) Peritoneal macrophage gene expression of anti-inflammatory macrophage marker: Chi3l3 and Mgl2. (**C**) Peritoneal macrophage gene expression of inflammatory cytokines: Il-1b, Il-6, Il-10, and Tnfa. All data are presented as mean ± SEM (n = 6). Groups that do not share the same letter are significantly different (*p* < 0.05) according to two-way ANOVA followed by Tukey’s multiple comparison post-hoc analysis. **Abbreviations**: CON: Control diet (45% calories from fat), HT: CON diet and received with daily HT intervention for 30 min. FO: isocaloric diet containing 10% fish oil, FOHT: FO diet and received daily HT intervention for 30 min, LPS: Lipopolysaccharide.

To delve into the effect of FO and HT on inflammation in OVX mice, we used acute LPS injection as the inflammation model. The peritoneal macrophage gene expression results showed that in LPS-injected mice, HT in both CON and FO diets significantly reduced proinflammatory macrophage marker monocyte chemokine protein 1 (*Mcp1*) (Figure 5A) and potently reduced inflammatory cytokine interleukin-1-beta (Il-1b) expression (Figure 5C). FO with HT had a more substantial inflammation reduction than HT alone. FO with HT did not have a significant impact on M2 anti-inflammatory markers chitinase-like 3 (*Chi3l3*) and macrophage galactose N-acetyl-galactosamine specific lectin 2 (*Mgl2*) (Figure 5B). There was no significant difference in interleukin-6 (*Il-6*), *Il-10*, and *Tnfa* among LPS and non-LPS injected mice or the treatment FO and HT effect.

## 4. Discussion

In this study, we investigated the effects of FO and HT on the metabolic health and gut microbiota of ovariectomized (OVX) mice. Consistent with our previous findings in aged female mice, reduced plasma lactate dehydrogenase (LDH) levels in the FO and HT groups indicated that HT alone or combined with FO did not cause cell damage. Our results also demonstrated that the combination of FO and HT significantly reduced body weight (Figure 1B) and improved insulin sensitivity (Figure 2). Gut microbiota analysis (Figure 4, Figures S1 and S2) revealed that FO and HT increased alpha-diversity and enriched beneficial microbial taxa, including *Eubacterium coprostanoligenes* and *Bacteroides*, which likely contributed to the observed improvements in insulin sensitivity and systemic inflammation. Our hyperthermia process involves repeated daily heat exposure for 30 min. In response to heat acclimation, we observed a decrease in resting core body temperature (Figure 1D). This effect is independent of FO intake but is reminiscent of the negative correlation between physical exercise intensity and core body temperature [33]. We speculate that HT-mediated thermoregulation is part of a feedback mechanism that enhances heat tolerance. Our findings suggest that HT-associated temperature adaptation could serve as a potential link to modulate gut profiles, thereby mitigating aging-related metabolic decline and immune susceptibility.

Additionally, FO and HT reduced inflammation, as evidenced by decreased levels of inflammatory markers such as *Mcp1* and *Il-1β* in the peritoneal macrophages upon LPS challenge. This anti-inflammatory response is particularly relevant, given the central role of inflammation in metabolic dysfunctions. Interestingly, the metabolic advantages of FO and HT did not extend to all aspects of lipid metabolism in iWAT, nor did they substantially impact hepatic lipid metabolism or thermogenesis. These tissue-specific effects suggest that FO and HT may act through mechanisms independent of lipid metabolism in certain tissues.

The metabolic parameters of plasma triglycerides and core body temperature were significantly correlated with specific bacterial taxa, such as *Alistipes* and *Coprostanoligenes group* (Figure S2), underscoring the potential role of gut microbiota in shaping host metabolism. 

Taken together, these results indicate that FO and HT could enhance metabolic health and decrease weight gain in menopause-related obesity in the absence of estrogen. Combining hyperthermia therapy and dietary interventions provides synergistic benefits that exceed those of either approach alone. HT has been reported to enhance metabolic flexibility, stimulate thermogenesis, and mimic some benefits of physical exercise [34,35]. These effects, coupled with FO’s anti-inflammatory and gut-modulating properties, suggest a synergistic potential for these interventions to act on complementary pathways to improve metabolic health and mitigate menopause-associated dysfunctions. A recent study by Lee et al. showed that combining FO with resistance exercise for 8 weeks improved cardiometabolic markers in post-menopausal women, including reduced blood pressure, triglycerides, and inflammatory and oxidative stress. These findings underscore the potential synergistic effects of FO and lifestyle interventions in mitigating metabolic dysfunctions associated with menopause [36]. Similarly, a study in 2022 reported that 12 weeks of FO with resistance exercise improved muscle strength, resting metabolic rate, and adipose tissue metabolism while reducing markers of systemic inflammation in older adults [37]. In our study, this synergy was evident in the significant improvements in insulin sensitivity, body weight regulation, and inflammatory markers observed in the FOHT group compared to individual treatments. The modulation of gut microbiota, including an increased abundance of beneficial taxa like *Bacteroides*, likely plays a critical mediating role, as these microbes are known to produce short-chain fatty acids that regulate energy metabolism and immune function [38].

Despite the promising results, there are several limitations. While the OVX mouse model is widely used to mimic post-menopausal estrogen deficiency, young OVX mice do not fully replicate the combined physiological changes associated with ovarian senescence in humans. In this context, alternative menopause models, such as those involving ovarian follicle depletion, may serve as better models for investigating the potential benefits of HT and FO in menopause-related metabolic dysregulation. Nonetheless, this study provided valuable insights into the specific metabolic and inflammatory consequences of estrogen loss independent of the aging process. The findings highlight the critical role of estrogen in regulating key metabolic pathways and gut microbiota, which are disrupted in its absence.

To better understand the relationship between intestinal microbiota and metabolic health during HT, as well as to explore the potential long-term effects of combined FO and HT interventions, additional research is essential. Future studies involving human participants are required to confirm the effects of HT and to determine the optimal frequency and intensity for different age groups. Evidence from human studies will provide valuable insights into the potential of hyperthermia treatment as a safe and effective strategy to reduce the risk of obesity and metabolic diseases, particularly for the aging population and individuals who may find it challenging to engage in regular physical activities. Specifically, randomized controlled clinical trials should be conducted with menopausal and older adults to assess the benefits and potential risks. Additionally, including male participants and non-aging individuals in clinical trials will allow for a more comprehensive understanding of the broader metabolic effects of hyperthermia therapy and fish oil supplementation.

## 5. Conclusions

In summary, this study demonstrated that combining fish oil (FO) supplementation and daily hyperthermia treatment (HT) improved metabolic and insulin sensitivity by protecting against inflammation and modulation of gut microbiota in the ovariectomized mouse model. These findings suggest that FO and HT could serve as effective interventions to address menopause-associated obesity and metabolic dysregulations.

## Figures and Tables

**Table 1:** **Blood biochemistry.** Plasma measurements of total cholesterol, triglycerides, plasma glucose, and Follicle-stimulating hormone.

	CON	HT	FO	FOHT
Total cholesterol (mg/dL)	217.8 ± 9.2 ^a^	230.8 ±9.4 ^a^	158.3 ± 18.2 ^b^	174.0 ± 17.7 ^ab^
Triglycerides (mM)	0.72 ± 0.10	0.62 ± 0.06	0.66 ±0.08	0.58 ± 0.05
Plasma glucose (mg/dL)	206.5 ± 5.5 ^a^	163.3± 3.5 ^c^	188.1 ± 4.3 ^b^	157.4 ± 3.0 ^c^
FSH (ng/mL)	4.3 ± 0.5	4.3 ± 0.5	4.6 ±1.3	3.9 ± 0.6

Values are presented as mean ± SEM (n = 6 per group). Groups that do not share the same letter (a–c) are significantly different (*p*-value < 0.05) according to one-way ANOVA with Tukey’s post-hoc multiple comparisons. FSH: Follicle-stimulating hormone.

## Data Availability

Data are available upon reasonable request to the corresponding author.

## Supplementary Materials

The following supporting information can be downloaded at https://www.mdpi.com/article/10.3390/nu17030549/s1. Figure S1. Microbiota beta-diversity in OVX mice compared to non-OVX mice; Figure S2. Correlation heatmap between gut microbiota and host metabolic parameters in CON and FOHT in OVX mice; Table S1. Diet composition table; Table S2. Primer sequences for RT-qPCR.
