# Supplementary material for: Effects of Fish Oil with Heat Treatment on Obesity, Inflammation, and Gut Microbiota in Ovariectomized Mice
Source: Nutrients. 2025 Jan 31;17(3):549. doi: 10.3390/nu17030549 (PMC11820760; doi:10.3390/nu17030549)
Supplement: Supplementary file 1 [file nutrients-17-00549-s001.zip › nutrients-3417252-supplementary.pdf]

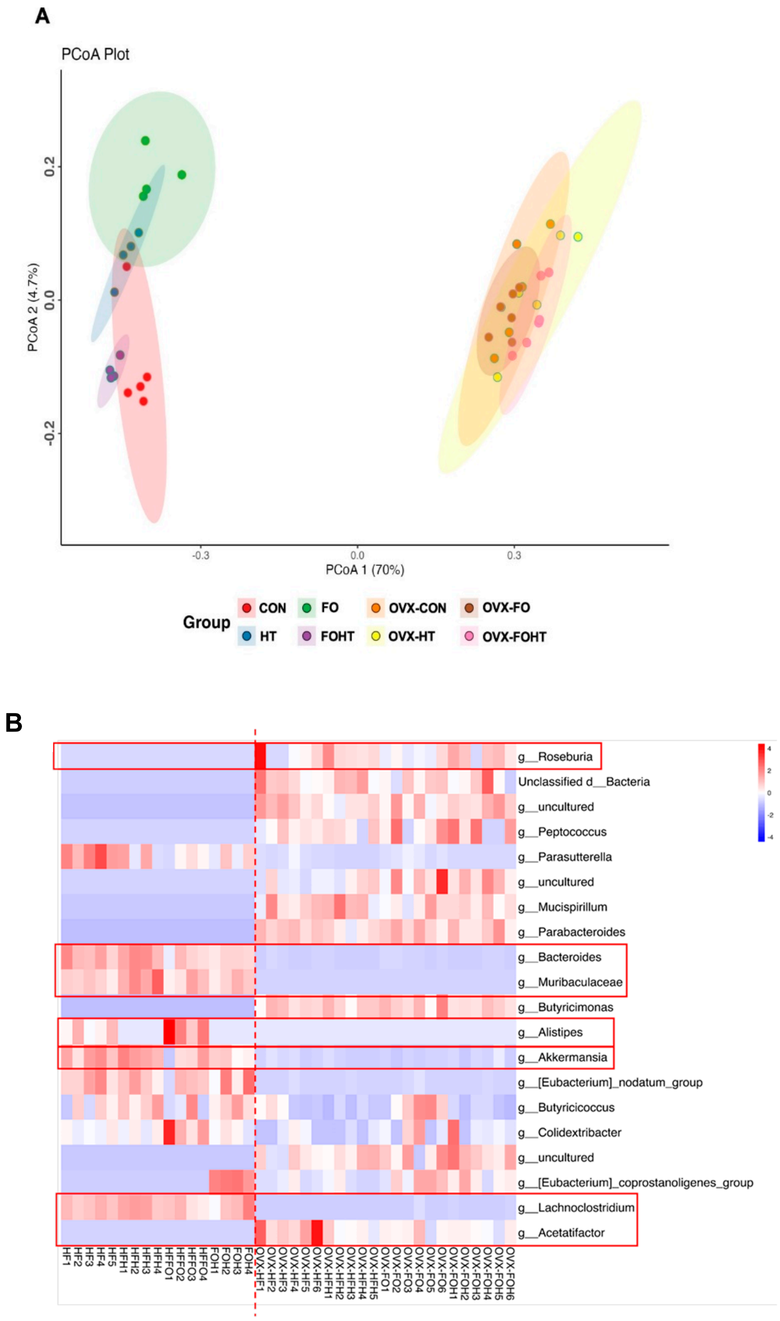

Figure S1. Microbiota beta-diversity in OVX mice compared to non-OVX mice. (A) Beta-diversity by PCoA based on the Bray-Curtis index distance and permutational multivariate analysis of variance (PERMANOVA). (B) Heatmap to show the gut microbes changes in the presence and absence of estrogen in the genus level. CON: control high-fat diet, HT: Hyperthermia treatment, FO: isocaloric fish oil containing high-fat diet; FOHT: isocaloric FO containing high-fat diet with hyperthermia treatment. OVX: ovariectomized mice.

A

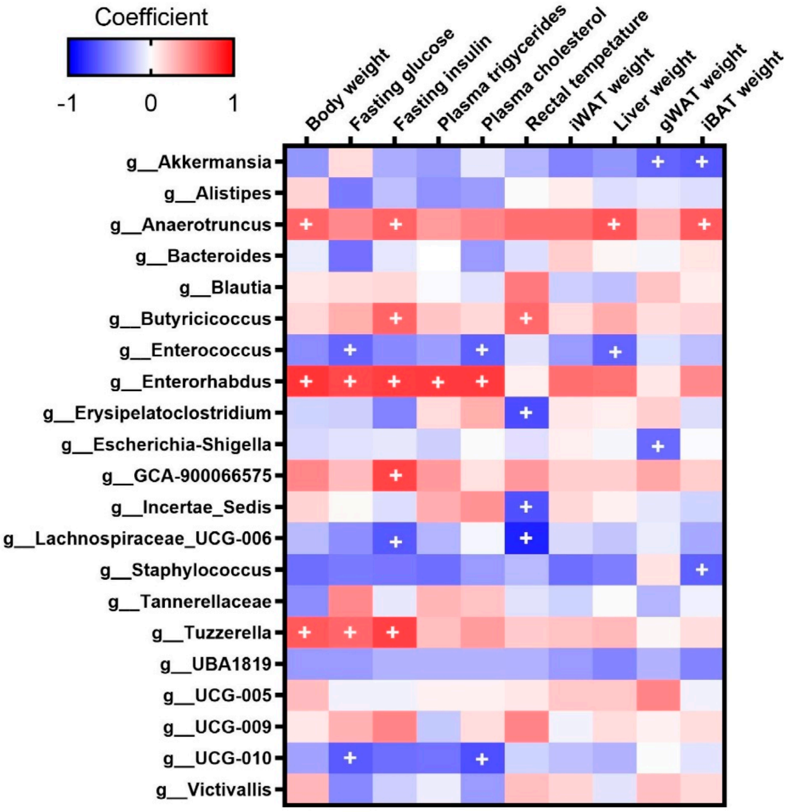

Figure S2. Correlation heatmap between gut microbiota and host metabolic parameters in CON and FOHT in OVX mice. Spearman ranked the correlation matrix of genus taxa abundance with host metabolic factors, body weight, fasting glucose, plasma triglycerides, food intake, rectal temperature, organ weights, and liver TG. "+" indicates statistically significant ($p<0.05$). The coefficient ranges (-1,1), the negative coefficient indicated a negative correlation, while the positive coefficient indicated a positive correlation.

Table S1. Diet composition table

Ingredients (g)	CON	FO
Casein	235	235
L-cystine	5.5	5.5
Corn starch	212	212
Maltodextrin	100	100
Sucrose	100	100
Palm oil	200	100
Fish oil	0	100
Soybean oil	40	40
Cellulose	50	50
Cholesterol	2	2
Mineral mix	35	35
Calcium carbonate	30	30
Vitamin mix	10	10
Choline bitartrate	2.5	2.5
% calories from Fat	45.28	45.28
% calories from CHO	34.55	34.55
% calories from Protein	20.17	20.17
Kcal/g diet	4.6	4.6

Table S2. Primer sequences for RT-qPCR

Gene	Forward/Reverse	Sequence (5′-3’)
mCpt1	Forward	CCAGGCTACAGTGGGACATT
	Reverse	AAGGAATGCAGGTCCACATC
mPpara	Forward	ACGATGCTGTCCTCCTTGATG
	Reverse	GTGTGATAAAGCCATTGCCGT
mScd1	Forward	GGGACAGATATGGTGTGAAACTATG
	Reverse	TTACAGACACTGCCCCTCAAC
mSrebp1c	Forward	GTGAGCCTGACAAGCAATCA
	Reverse	GGTGCCTACAGAGCAAGAGG
mFas	Forward	GGAGGTGGTGATAGCCGGTAT
	Reverse	TGGGTAATCCATAGAGCCCAG
mDagt1	Forward	TCCGCCTCTGGGCATTC
	Reverse	GAATCGGCCCACAATCCA
mMcp1	Forward	AGGTCCCTGTCATGCTTCTG
	Reverse	GCTGCTGGTGATCCTCTTGT
mF4/80	Forward	CTTTGGCTATGGGCTTCCAGTC
	Reverse	GCAAGGAGGACAGAGTTTATCGTG
mTnfa	Forward	GGCTGCCCCGACTACGT
	Reverse	ACTTTCTCCTGGTATGAGATAGCAAAT
mTrpv1	Forward	CATCTTCACCACGGCTGCTTAC
	Reverse	CAGACAGGATCTCTCCAGTGAC
mSerca2b	Forward	ACCTTTGCCGCTCATTTTCCAG

	Reverse	AGGCTGCACACACTCTTTACC
mAtp2b1	Forward	GCACAGTCTCAGAGCAACGACA
	Reverse	GCCACATCAGTTCCAGCAATGC
mAtp2b2	Forward	CGGATAAGCACACGCTGGTCAA
	Reverse	AGCCCACATCTGCCTTCTTGAG
mSlc8a	Forward	GGACCAGTTCATGGAAGCCATC
	Reverse	CACAGGCAAAGAGCACCTTCCA
m36b4	Forward	GGATCTGCTGCATCTGCTTG
	Reverse	GGCGACCTGGAAGTCCAACT
mUCP1	Forward	AGGCTTCCAGTACCATTAGGT
	Reverse	CTGAGTGAGGCAAAGCTGATTT
mMcp1	Forward	GCTACAAGAGGATCACCAGCAG
	Reverse	GTCTGGACCCATTCCTTCTTGG
mCd11c	Forward	CTGGATAGCCTTTCTTCTGCTG
	Reverse	GCACACTGTGTCCGAACTC
mChi3l3	Forward	AGAAGGGAGTTTCAAACCTGGT
	Reverse	GTCTTGCTCATGTGTGTAAGTGA
mMgl2	Forward	TTAGCCAATGTGCTTAGCTGG
	Reverse	GGCCTCCAATTCTTGAAACCT
mIl-1b	Forward	GTCACAAGAAACCATGGCACAT
	Reverse	GCCCATCAGAGGCAAGGA
mIl-6	Forward	CTGCAAGAGACTTCCATCCAGTT
	Reverse	AGGGAAGGCCGTGGTTGT

mIl-10	Forward	GCTCTTACTGACTGGCATGAG
	Reverse	CGCAGCTCTAGGAGCATGTG
